# Pilot study characterizing a single pooled preparation of equine platelet lysate for nebulization in the horse

**DOI:** 10.3389/fvets.2024.1488942

**Published:** 2024-12-12

**Authors:** Patricia Egli, Lindsey Boone, Laura Huber, Courtney Higgins, Pankaj P. Gaonkar, Justine Arrington, Maria C. Naskou, John Peroni, Julie Gordon, Kara M. Lascola

**Affiliations:** ^1^Department of Clinical Sciences, College of Veterinary Medicine, Auburn University, Auburn, AL, United States; ^2^Department of Pathobiology, College of Veterinary Medicine, Auburn University, Auburn, AL, United States; ^3^Roy J. Carver Biotechnology Center, Proteomics Core, University of Illinois, Urbana, IL, United States; ^4^JF Peroni Laboratory, Department of Large Animal Medicine, College of Veterinary Medicine, University of Georgia, Athens, GA, United States

**Keywords:** equine, platelet lysate, respiratory, nebulization, antibiotic alternative

## Abstract

**Introduction:**

Platelet lysate (PL) demonstrates antimicrobial and anti-inflammatory properties offering potential for treatment of bacterial pneumonia in horses. It remains unknown whether nebulization is suitable for PL administration in horses. This pilot study characterized particle size and flow rate of pooled equine PL (single preparation) nebulized using an equine-specific nebulizer (Flexivent^®^).

**Methods:**

Protein composition and antimicrobial activity were compared before and after nebulization. Protein composition was evaluated according to growth factor, antimicrobial peptide and cytokine concentrations and proteomic analysis. To evaluate antimicrobial activity, bacterial growth inhibition [maximum growth (μmax); carrying capacity (K)] were determined for *E. coli, Streptococcus equi* subsp *zooepidemicus* and *Rhodococcus equi* (WT and MDR) using pre- and post-nebulized PL concentrations of 50%.

**Results:**

Flow rate and median particle size were 0.8 ml/min and 4.991 μm with 52% of particles ≤ 5 μm. Differences in PL protein composition were detected with nebulization. For *E. coli* and *S. zooepidemicus*, nebulization did not alter effect of PL on growth parameters. PL treatments decreased K for *S. zooepidemicus* (*p* = 0.009) compared to BHI. For *R. equi* K was increased post- vs. pre-nebulization (WT and MDR) and μmax increased pre- vs, post-nebulization (MDR). PL treatments increased K and μmax for MDR *R. equi* and μmax for WT *R. equi* compared to BHI (*p* ≤ 0.05).

**Conclusion:**

Nebulization of PL *in vitro* is technically feasible. The results of this study support further investigation to better characterize the effect of nebulization on PL and its suitability for nebulization in horses.

## 1 Introduction

Bacterial bronchopneumonia represents a significant cause of morbidity and loss of performance in horses of all ages. In adult horses and older foals, bronchopneumonia most often develops secondary to colonization by opportunistic bacteria from the upper respiratory tract and oral cavity into the lower airways with *Streptococcus equi* subsp *zooepidemicus* remaining the most frequently isolated bacterium (1). In nursing and weanling foals, bronchopneumonia caused by the primary respiratory pathogen *Rhodococcus equi*, is of worldwide significance (2–4). Antimicrobial medications represent the mainstay of treatment for bronchopneumonia in horses, regardless of the pathogen (1, 2). Unfortunately, antimicrobial resistance is an ever-growing global concern and resistance of bacterial pathogens associated with equine pneumonia, including *Streptococcus equi* subsp *zooepidemicus* and *Rhodococcus equi*, is reported (5–8). Concern of new and evolving antimicrobial resistance patterns, including multi-drug resistance, has also been raised for these pathogens (6). For example, antimicrobial and multi-drug resistance has markedly increased for *Rhodococcus equi* over the past decade (3, 4). Novel therapies are needed to reduce antimicrobial use and/or improve therapeutic potential of existing antimicrobials for treatment of bacterial bronchopneumonia in horses.

Traditionally, platelets have been recognized for their role in hemostasis and wound healing. More recently they have received greater recognition for their contribution to multiple and diverse regenerative, immunologic, and anti-inflammatory processes (9). Many of these non-hemostatic functions are derived from biologically active molecules stored and released in platelet α granules upon platelet activation. These bioactive molecules include growth factors as well as immunologic, anti-inflammatory, and antimicrobial molecules concentrated at supraphysiologic levels in platelet derived biotherapeutics, platelet lysate (PL) and platelet-rich-plasma (PRP) (10–13). Both PL and PRP have received increased recognition in human and veterinary medicine due to their anti-inflammatory, immunomodulatory, and antimicrobial properties (10–16). The composition, processing and disease modifying effects of PRP and PL in veterinary medicine have been reviewed recently (14, 17, 18). PL is prepared by activating platelets in PRP or concentrates obtained through plateletpheresis followed by filtration to remove platelet membranes (10, 19). Lysis of the platelets during preparation of PL releases the bioactive molecules and confers additional advantages over PRP, including preparation as an allogenic (vs. autologous), acellular product that can be frozen and stored for use on an as-needed basis (10, 12, 19). Furthermore, pooled PL (i.e., derived from platelet concentrates from multiple horses) demonstrates both reduced variability and increased concentrations of these bioactive molecules (10, 12).

Therapeutic investigation of PL, as well as PRP, in veterinary medicine has focused on their anti-inflammatory and regenerative potential for musculoskeletal injury as well as bacterial infections (10, 20). It is through its anti-inflammatory and antimicrobial potential that PL may offer promise for the treatment of respiratory disease in horses. Bioactive molecules considered important for anti-inflammatory and immunomodulatory activity of PL include various cytokines, chemokines, and growth factors important in mitigating inflammatory cell recruitment and dendritic cell response (9). Peptides and small proteins present in PL with potential antimicrobial activity include defensins, thrombocidins, thymocin β4, RANTES, complement precursors, and kinocidins, as well as oxygen metabolites (9, 11, 21). While the exact mechanisms directing antimicrobial activity of PL are ill-defined, it appears to demonstrate primarily bacteriostatic activity against a variety of gram negative and positive bacterial organisms including those of direct relevance to respiratory disease in horses, such as *S. equi* subsp *zooepidemicus* (10–12) and may also enhance activity of antimicrobials when administered concurrently (20).

Limited information is available regarding the therapeutic potential of PL or other platelet derived biotherapeutics for the treatment of respiratory disease (22). Experimental studies in mice administered intraperitoneal PL demonstrated improved lung vascular and alveolar regeneration post-pneumonectomy as well as reductions in vascular damage after LPS challenge and in a separate study, inhibition of airway hyperresponsiveness and airway eosinophilia in a murine asthma model (23, 24). Evaluation of PL for the treatment of musculoskeletal pathology demonstrates that local administration at the affected site is important for therapeutic efficacy (12, 14, 25–27). While intrabronchial instillation of platelet rich plasma (PRP) has been described in horses diagnosed with equine asthma (28) and exercise induced pulmonary hemorrhage (29), nebulized administration could provide a superior and non-invasive method to deliver PL directly to the lower airways in horses suffering from bronchopneumonia or other respiratory diseases. A single study demonstrated that nebulization of PRP was well-tolerated in a small group of humans suffering from inhalation lung injury (30). A separate *ex vivo* study suggests that growth factor concentrations and cell growth in culture containing human PL are maintained after nebulization (22).

The impact of nebulization on protein stability and bioactivity of protein molecules has been documented (31) but varies according to type of nebulizer and specific protein content of a solution. Given the complex protein composition of PL and unknown species related differences, evaluating species-specific and nebulizer-specific impacts on protein composition and bioactivity for PL is essential. Furthermore, given the viscous nature of PL (19) determining whether nebulization generates particles of suitable size to reach the lower airways is of direct clinical relevance. Thus, this pilot study serves as an important first step toward determining the potential clinical application of nebulization of equine PL for treatment of bacterial bronchopneumonia in horses. Our specific objectives were to characterize particle size and flow rate of a single pooled preparation of equine PL nebulized using a commercially available equine mesh nebulizer (Flexivent^®^), and to also evaluate and compare growth factor, cytokine, and protein composition of PL as well as antimicrobial activity against *S. equi* subsp *zooepidemicus, R. equi*, and *E. coli*, before and after nebulization. We predicted that clinically relevant flow rates (0.5–1 mL/min) and aerosolized particle sizes (< 10 μm) would be achieved, and that PL growth factor, cytokine and protein composition and antimicrobial activity would remain stable over the process of nebulization.

## 2 Materials and methods

### 2.1 Manufacture of pooled PL

Pooled equine PL was manufactured at the University of Georgia (JF Peroni laboratory, IACUC approval #A2018 01-013) as previously described (12). Briefly, one liter of platelet concentrates obtained via plateletpheresis from three mixed-breed, healthy adult university-owned horses (aged 4–14 years) were subject to two freeze-thaw cycles to lyse the platelets and release their contents (28). Each was then centrifuged three times, filtered through a Falcon cell strainer (40 μm) and Stericup sterile vacuum filter with cellulose acetate membrane (0.45 μm) to remove cellular debris and then pooled and centrifuged (30 min × 2,800 g) to insure thorough mixing. An aliquot of the pooled product was subjected to fungal and bacterial culture and the remainder stored at −80°C as 50 mL aliquots.

### 2.2 Nebulization of PL

**Figure 1:**
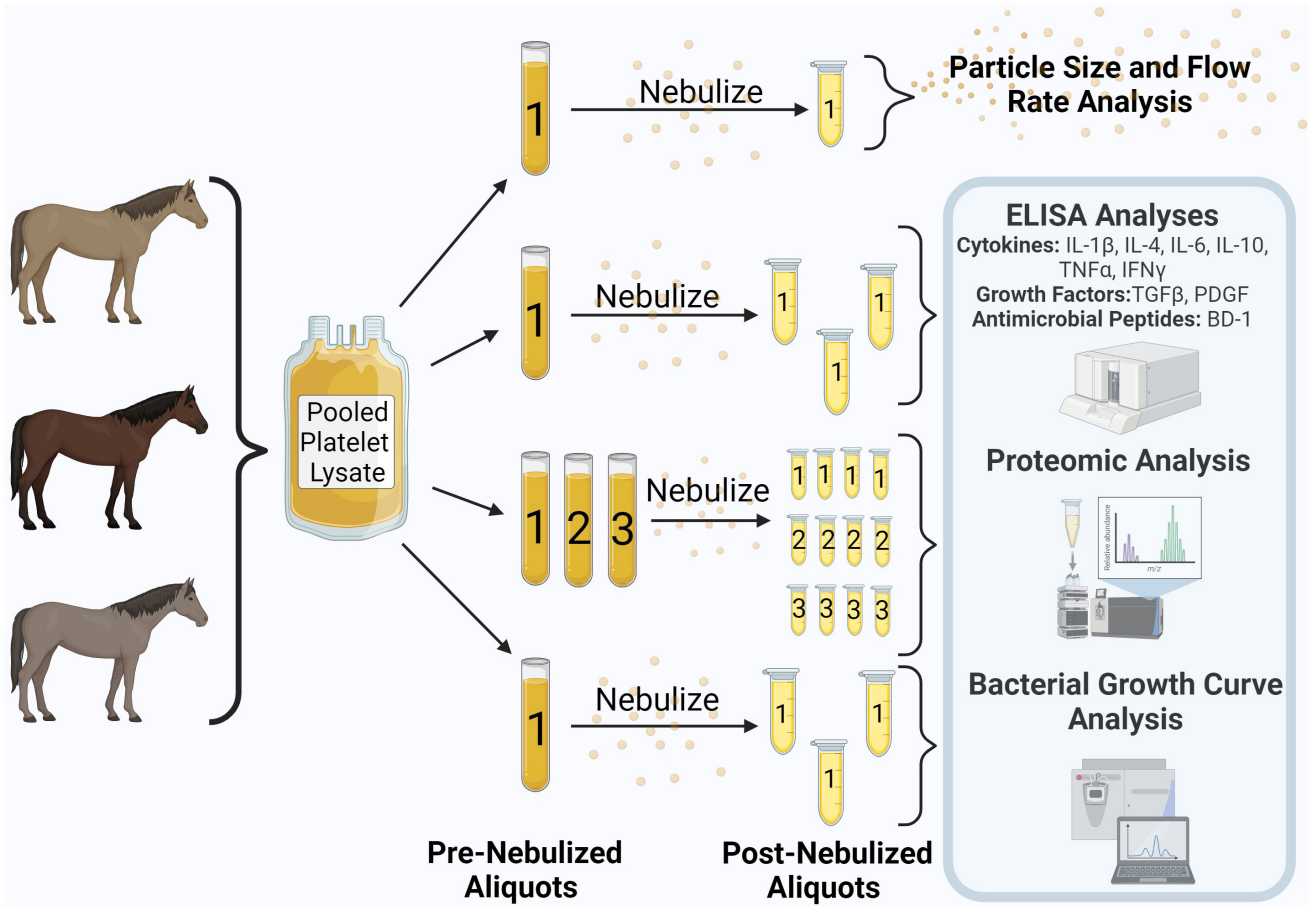
Study design. Equine pooled platelet lysate was aliquoted into pre-nebulization samples. Samples underwent nebulization and were collected into additional aliquots. Analyses contained within the blue shaded box were performed on both the pre- and post-nebulized samples. This figure was created using Biorender.com.

For experimental purposes, 50 mL of PL was thawed in a 37°C water bath and then centrifuged for 30 min at 2,800 × g. A single 10 mL aliquot was separated and stored at −80°C for later measurement of flow rate and aerosol particle size. The remainder of the PL was divided into aliquots for pre- and post-nebulization comparisons (Figure 1). Aliquots that were not to be subjected to nebulization were stored at −80°C until analysis for comparison with post-nebulized samples. Aliquots allotted for nebulization were nebulized within 2 months of freezing and all further analysis was performed within a 9-month period. Nebulization of PL using a commercially available equine mesh nebulizer (Flexineb E3^®^, Union City, TN, USA) equipped with a standard (gray, Standard Flow Medication) cup was performed within a laminar flow hood and under sterile conditions. Nebulized PL condensate was collected into a sterile conical tube, aliquoted, and stored at −80°C for later analysis. A single aliquot underwent nebulization for cytokine, growth factor, and antimicrobial peptide measurement, three aliquots underwent separate nebulization for proteomic analysis, and a single aliquot underwent nebulization for *in vitro* bacterial growth curve analysis. A single medication cup was used for the study. According to manufacturer instructions (Nortev Limited, Claregalway, Galway, Ireland) the cup was cleaned between each nebulization and was additionally cold sterilized prior to nebulization of samples for growth curve analysis.

#### 2.2.1 Flow rate and aerosol particle size measurement

Flow rates and aerosol particle size were measured by the manufacturer using a spray particle and spray droplet size analyzer (Spraytec; Malvern Panalytical Ltd, Malvern, UK) equipped with a standard medication cup (32, 33). For measurement of flow rate, saline was used as the standard for comparison. Particle size measurements were taken every 3 s over a 60 s interval and reported as minimum and maximum observed particle size, volume mean diameter 50 (DV50), and percent measured particles below 5 and 10 μm, respectively.

### 2.3 Measurement of cytokines, growth factors and Beta Defensin-1

Concentrations of IL-1β, IL-4, IL-6, IL-10, TNF-α, IFN-γ were measured on single pre- and single post-nebulized PL aliquots using a multiplex bead immunoassay (Equine Cytokine Magnetic Bead Panel, Millipore Sigma, Burlington, MA, USA) according to the manufacturer's instructions (34). All samples were analyzed in triplicate using a 96-well platform. A minimum bead count of 50 for each cytokine was acquired for data analysis. Data were analyzed using xPONENT^®^ Immunoassay Curve Fitting Software from Luminex, using the MAGPIX^®^ analyzer (Luminex Corporation, Austin, TX, USA). For each cytokine, the average concentration across triplicate measurements was calculated and used to calculate percent increase or decrease between pre- and post-nebulized PL samples.

ELISA analysis was performed on single pre- and single post-nebulized PL aliquots using commercially available kits previously validated in horses for TGF-β1 and PDGF (Human PDGF-BB DuoSet DY220 and Human TGF-β_1_ DuoSet, DY240E, R&D Systems, Minneapolis, MN) and for Beta Defensin-1 (BD-1) antimicrobial peptide (Nori^®^ equine Beta-Defensin1, Genorise Scientific, INC, Glen Mills, PA, USA). Standards were provided for all kits and used to prepare standard curves according to manufacturer instructions (35–37). Samples were performed in triplicate (TGF-β1, BD-1) or duplicate (PDGF) and were diluted to 1:20. Average concentrations across replicate measurements were calculated and used to calculate percent increase or decrease between pre- and post-nebulized PL samples.

### 2.4 Proteomic analysis

Proteomic profiles on the single pre- and three post- PL samples were evaluated using high resolution LC-MS. The PL samples underwent protein digestion for protein identification or ultrafiltration for endogenous peptide identification prior to mass spectrometric analysis. For all methods for protein identification, unless otherwise specified, chemicals were purchased from Sigma. Optima grade LC-MS mobile phases were purchased from Fisher Scientific (Waltham, MA, USA). Ultrapure water (18.2 MΩ-cm) was obtained from a Barnstead Nanopure system.

#### 2.4.1 Sample preparation with digestion for total protein identification

Platelet lysate samples were denatured in a buffer of 4 M GuHCl in 100 mM TEAB. Next, 10 mM TCEP and 40 mM CAA (TCI) were added, and the samples were heated to 95°C for 10 min to promote reduction of disulfide bonds and alkylation of cysteine residues. The proteins were then precipitated with chloroform and methanol, and the resulting pellets were briefly air dried. The protein pellets were dissolved in 2 M GuHCl in 50 mM TEAB and digested for 1 h with 2.5 μg LysC (Fujifilm Wako Chemicals, Richmond, VA, USA). After the pellets were fully dissolved, a BCA assay (Pierce, Fisher Scientific) was used to check protein concentration. Additional LysC was added to a final concentration of 1:100 w/w (enzyme:substrate), and samples were incubated at 25°C for 3 h. The samples were further diluted with 50 mM TEAB to GuHCl < 1M and digested overnight with trypsin (Pierce, Fisher Scientific) 1:50 w/w at 37°C. On the following day, the digested peptides were acidified with 10% TFA, desalted with StageTips (29), and dried in a vacuum centrifuge. The peptides were suspended in 5% ACN in 0.1% FA to a final concentration of 200 ng/μL, as determined by a colorimetric peptide BCA assay (Pierce).

#### 2.4.2 Sample preparation with ultrafiltration for endogenous peptide identification

Platelet lysate samples were similarly denatured as described above for protein digestion, except that after alkylation of cysteine residues the samples were cooled and passed through Amicon 0.5 mL 30 K MWCO ultra centrifugal filters for a total of 20 min at 14,000 × g. The filters were washed with 200 μL of water and centrifuged for another 20 min at 14,000 × g; this wash was added to the initial flow-through. The peptides and small proteins that passed through the ultra-centrifugal filters were acidified with 10% TFA, desalted with StageTips (38), and dried in a vacuum centrifuge. The peptides were then suspended in 5% ACN in 0.1% FA to a final concentration of 500 ng/μL, as determined by a colorimetric peptide BCA assay (Pierce).

#### 2.4.3 LC-MS analysis digested lysate

Each sample (400 ng) was analyzed by an UltiMate 3000 RSLCnano system coupled to an Orbitrap Fusion mass spectrometer (Thermo Fisher, Waltham, MA, USA). The LC was equipped with a μPAC 315 μm × 50 cm C18 column (Thermo Fisher) and operated at a flow rate of 300 nL/min with mobile phases of 0.1% FA (A) and 0.1% FA in 80% ACN (B). The peptides were separated with a gradient of 12–36% B over 120 min, which was followed by column cleaning and equilibration. The mass spectrometer was run in positive mode for data dependent acquisition. Precursor scans using the orbitrap analyzer were collected at 120 k resolution with a scan range of 300–2,000 *m/z* (max IT 100 ms, normalized AGC target 50%). Additional settings included exclusion of charge states ≥8 and a dynamic exclusion window of 60 s. MS2 spectra were collected in the ion trap in rapid mode after CID fragmentation (35% fixed energy). An isolation window of 1.6 *m/z* was used with a total DDA cycle time of 3 s.

#### 2.4.4 LC-MS analysis filtered lysate

Each sample (500 ng) was then analyzed by an UltiMate 3000 RSLCnano system coupled to a Q Exactive HF-X mass spectrometer (Thermo Fisher). The LC was operated at a flow rate of 300 nL/min with mobile phases of 0.1% FA (A) and 0.1% FA in 80% ACN (B). The peptides were separated with a 25 cm Acclaim PepMap 100 C18 column (2 μm particle size, 75 μm ID) maintained at 50°C over the course of the run. The gradient was 5–35% B over 90 min and then 35–50% B over 5 min, followed by column washing and equilibration. The mass spectrometer was operated in positive polarity with MS1 scans from 350 to 2,000 *m/z* at 120 k resolution (50 ms max IT; 3e6 AGC) followed by HCD fragmentation (30 NCE) of the top 15 most abundant ions. MS2 scans were collected at 15 k resolution with an isolation window of 1.0 *m/z*, a maximum IT of 30 ms, and an AGC target of 5e4. Unassigned and singly charged ions were excluded from selection for MS2, and the dynamic exclusion window was 45 s.

#### 2.4.5 Protein identification and quantification of digested and filtered lysate

The raw LC-MS data was processed with Byonic v5.3.5 (Protein Metrics, Cupertino, CA, USA) implemented in Proteome Discoverer v2.4.1.15 (Thermo Fisher) to identify the proteins; label-free quantitation (LFQ) was done with Minora feature finder and the precursor ion quantifier nodes in Proteome Discoverer. Settings for the Byonic search included a precursor mass tolerance of 10 ppm and a fragment mass tolerance of 0.5 Da for digested lysate and 20 ppm for filtered lysate. For digested lysate, tryptic digest with a maximum of two missed cleavages was specified along with a fixed modification for cysteine carbamidomethylation and variable modifications of protein N-terminal acetylation, methionine oxidation, and asparagine/glutamine deamidation. For filtered lysate, no enzyme was specified for digestion, and a fixed modification for cysteine carbamidomethylation and variable modifications of protein N-terminal acetylation and methionine oxidation were added to the searches. The LC-MS data were deposited with the ProteomeXchange Consortium via the jPOST partner repository (https://jpostdb.org) with the dataset identifier PXD055023.

For both digested and filtered lysate, searches were done against the Uniprot *Equus caballus* reference proteome (69,389 entries; downloaded October 2023). Using a reverse decoy database strategy, the false discovery rate (FDR) was set to 1%. For LFQ, a maximum RT shift of 10 min was allowed for mapping features across files for digested lysate and 5 min was permitted for chromatographic alignment. For digested and filtered lysate, both unique and razor peptide intensities were used for quantitation, but shared peptides were used for quantitation of only one protein—the protein with the highest number of other identified peptides. Protein abundances were calculated from the sum of their peptide abundances. For filtered lysate, normalization by total peptide amount was performed prior to calculation of protein abundances with the protein abundance ratios determined from the median of all possible pairwise peptide comparisons.

### 2.5 Bacterial growth curve assay to determine inhibition of bacterial growth

Bacterial growth curve analysis was performed on *Rhodococcus equi, Escherichia coli* (ATCC^®^25922^TM^, Manassas, VA, USA), and *Streptococcus equi* subsp *zooepidemicus*. Clinical isolates included a *Rhodococcus equi* strain susceptible to standard antimicrobials (**WT-*R. equi***) and a multidrug resistant (**MDR-*R. equi***) strain (both provided by L. Huber laboratory, Auburn University, Auburn, AL, USA) as well as an equine respiratory isolate for *Streptococcus equi* subsp *zooepidemicus* (provided by the Clinical Microbiology Laboratory, Auburn University College of Veterinary Medicine, Auburn, AL, USA). All bacteria were cultured in BBL Brain Heart Infusion broth (BHI, Becton Dickson, Franklin Lakes, NJ, USA) at 37°C with shaking (200 rpm). The optical density of the bacterial solution was measured at 600 nm (OD_600_) using a BioRad SmartSpec Plus spectrophometer. The OD_600_ was adjusted to 0.02, corresponding to ~10^7^ CFU/mL (12). The required number of 1 mL aliquots were removed from each tube of bacterial solution and centrifuged at 3,000 rpm for 10 min to pellet the bacteria. Pellets were then washed with PBS and 500 μl of the appropriate treatment (50% pre-/post-nebulized PL diluted in BHI) or positive control (50% BHI diluted in PBS) was added. Corresponding negative controls (no bacteria added) for both treatment groups and 50% BHI were included for analysis. All treatments were prepared in duplicate and were transferred to a 48-well plate to be analyzed by an automated plate reader (Synergy HT, BioTek, Shoreline, WA, USA) measuring OD_600_ (corresponding to the number of bacteria in each well) every 10 min for 30 h. Blank no-bacteria were used to provide OD_600_ values for treatments only. Bacterial growth inhibitor parameters [maximum growth (μmax); carrying capacity (K)] were calculated for *Streptococcus equi* subsp *zooepidemicus, Escherichia coli*, and *Rhodococcus equi* (**WT-*R. equi*** and **MDR-*R. equi***) using pre- and post-nebulized PL concentrations of 50% (**PreN50**, **PostN50**).

### 2.6 Statistical analysis

In accordance with the limited number of pre- and post-nebulized replicates used for analysis in this pilot study, descriptive statistics were primarily used to present the data. For cytokine, growth factor, and Beta Defensin-1 the average concentrations across duplicate or triplicate measurements are reported and were used to calculate the percent increase or decrease between pre- (*n* = 1) and post-nebulized (*n* = 1) PL samples. Descriptive statistics were also used to present proteomics data and for comparisons between pre- (*n* = 1) and post-nebulized (*n* = 3) lysate samples. A cutoff ratio of >1.5 or < 0.5 was used to suggest meaningful differences in protein abundance between pre- and post-nebulized platelet lysate. Gene ontology (GO) analysis was performed to categorize platelet lysate according to GO-biological processes terms, GO-cellular components terms, and GO-molecular functions terms using the DAVID (Database for Annotation, Visualization, and Integrated Discovery) functional annotation tool (https://david.ncifcrf.gov/). For analysis of the bacterial growth curve assay, the R package “growth-rates” was used to estimate the maximum growth rate during exponential growth (μmax) and carrying capacity (K) from OD_600_ values. Linear modeling was used to evaluate the effect of conditions on μmax and K and multipair wise comparisons between conditions were performed using *post-hoc* tests (Tukey HSD test). Statistical significance was set at *p* ≤ 0.05. Normality of data was assessed by plot visualization. Statistical analyses were performed using RStudio (version 2023.12.1+402).

## 3 Results

### 3.1 Platelet lysate nebulized flow rate and aerosol particle size

The measured flow rate of PL during nebulization was 0.8 ml/min compared to a flow rate of 1 ml/min of saline. Minimum and maximum aerosolized particle diameters were 0.10 μm and 2,500 μm. The DV 50 was 4.991 μm, with 52% of aerosolized particles ≤ 5 μm in size and 85% of particles ≤ 10 μm in size.

### 3.2 Cytokine, growth factor and antimicrobial peptide concentrations in pre- and post-nebulized platelet lysate

Measured concentrations in the pre- (*n* = 1) and post-nebulized (*n* = 1) PL samples for cytokines, TGF-β1, PDGF, and BD-1 along with percent change in concentrations between pre- and post-nebulized samples are reported in Table 1. Relative to pre-nebulization, all measured concentrations were decreased in the post-nebulized sample, except for IL-4. These differences were < 10% for all measured variables excluding IL-1β (22%), TGF-β1 (20%), and BD-1 (16%).

**Table 1:** Pre- (*n* = 1 aliquot) and post-nebulized (*n* = 1 aliquot) platelet lysate concentrations and respective percent difference between the two conditions for cytokines (IL-1β, IL-4, IL-6, IL-10, TNF-α, IFN-γ), growth factors (TGF-β1, PDGF), and Beta Defensin-1 (BD-1).

	**Pre-nebulization (pg/ml)**	**Post-nebulization (pg/ml)**	**Change (%)**
**Cytokines**			
IL-1β	21.8	17	−22
IL-4	909	926	+1.8
IL-6	41.6	38.9	−6.7
IL-10	82.4	75.3	−8.6
TNF-α	14.5	13.4	−7.7
IFN-γ	640	597	−6.7
**Growth factors**			
TGF-β1	3,727	2,968	−20
PDGF	1,634	1,589	−2.7
**Antimicrobial peptide**			
BD-1	27,888	23,414	−16

### 3.3 Proteomic analysis

#### 3.3.1 Protein identification and characterization

**Figure 2:**
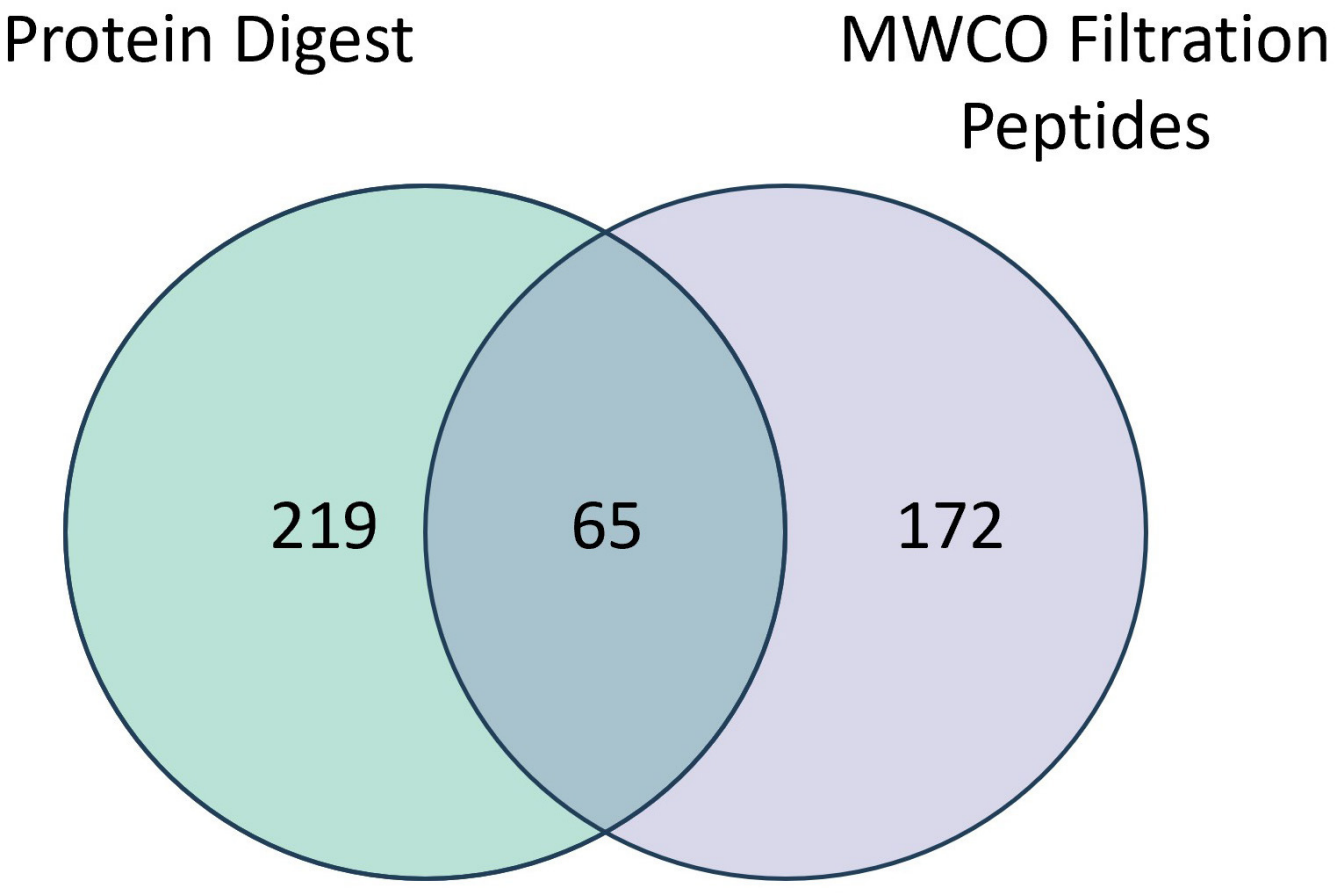
Venn diagram depicting overlap in proteins identified through digestion or filtration (MWCO) of pre-nebulized (*n* = 1) and post-nebulized (*n* = 3) platelet lysate samples.

A total of 456 proteins were identified in the pre- and post-nebulized platelet lysate pools using protein digestion and endogenous peptide identification through low molecular weight cut-off (MWCO) filtration. The number of proteins identified through digestion, MWCO filtration and those found common to both methods are presented in Figure 2 with a list of identified proteins available in Supplementary Table 1. Qualitatively, the most common groups of proteins (>20% of total proteins) identified across both methods include (in decreasing order of prevalence): complement system proteins, Ig like domain proteins, actin related proteins, tubulin related proteins, apolipoproteins, glycoproteins, coagulation factors, serpin family proteins, immunoglobulin component proteins, growth factors, and α-1 antitrypsin related proteins.

**Figure 3:**
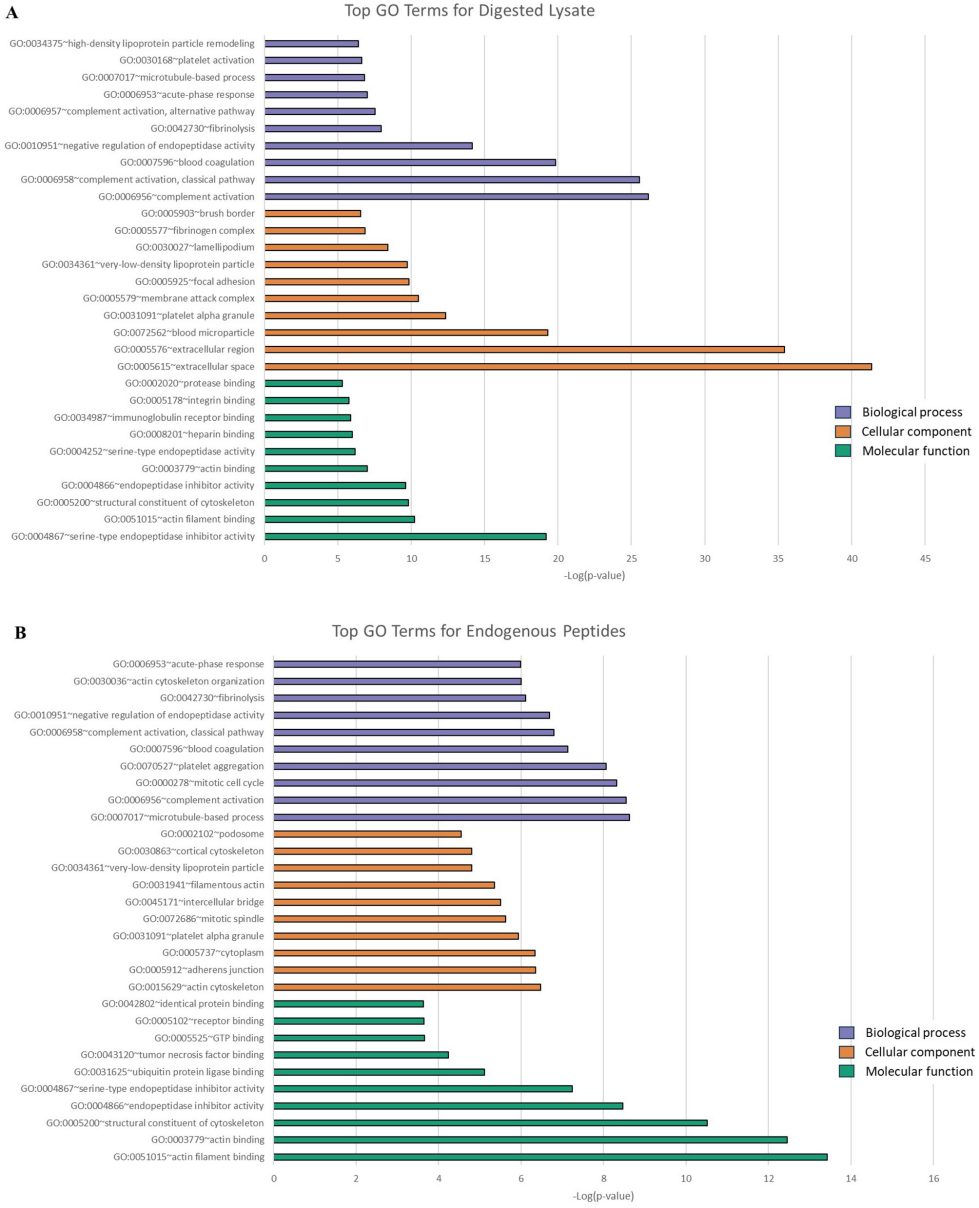
Categorization of digested **(A)** and filtered **(B)** equine platelet lysate by gene ontology (GO) according to the top ten GO terms for biological processes (purple), cellular components (orange), and molecular functions (green) using DAVID. The top ten terms chosen according to most significant *P*-values. Categorization based on pooled data from pre-nebulized (*n* = 1) and post-nebulized (*n* = 3) samples.

Go Analysis on the digested lysate (Figure 3A) identified GO terms for 252/284 proteins categorized according to molecular function (MF; *n* = 72 terms), 255/284 proteins categorized according to biological processes (BP; *n* = 166 terms) and 257/284 proteins categorized according to cellular content CC; *n* = 75 terms). Similarly, Go Analysis on MWCO lysate (Figure 3B) identified GO terms for 216/237 proteins categorized according to molecular function (MF; *n* = 55 terms), 225/237 proteins categorized according to biological processes (BP; *n* = 155 terms) and 227/237 proteins categorized according to cellular content (CC; *n* = 74 terms). The top 10 terms for each category based on *P*-value are presented in Figures 3A, B. The most common biological processes identified for PL included complement activation, blood coagulation, acute phase response, fibrinolysis, and endopeptidase regulation among others.

#### 3.3.2 Minor differences in protein composition were identified between pre- and post-nebulized platelet lysate for both digested and filtered lysate

A total of 281/284 protein groups identified through digestion were common to the pre- and post-nebulized PL samples. No proteins were unique to the pre-nebulized lysate. Three proteins (Ig-like domain containing protein and two Actin related proteins) were exclusive to post-nebulized samples but were not uniformly expressed across all samples. A total of 2,890 quantifiable peptides were identified through filtration representing 237 protein groups found in both the pre- and post- nebulized PL samples. Fourteen peptides were unique to the pre-nebulized sample. One of these, beta defensin 3, was found only in the pre-nebulized sample with the remaining peptides corresponding to proteins found in both pre- and post-nebulization samples. Thirty-three peptides were unique to the post-nebulized samples. These peptides corresponded to seven proteins exclusive to post-nebulized samples, with none uniformly expressed across all samples.

#### 3.3.3 Minor differences in protein and peptide abundance were identified between pre- and post-nebulized platelet lysate for both digested and filtered lysate

For the 281 proteins common to pre- and post-nebulized samples as identified through digestion, abundance was qualitatively similar between the pre- and post-nebulized samples. The median (IQR) abundance ratio for pre-nebulized sample/pooled post-nebulized samples was 1.034 (5.31–0.05). Using cutoff ratios of ≥1.5 or ≤ 0.5 to suggest differences in protein abundance, 7/281 proteins demonstrated increased abundance in the pre-nebulized sample and 12 proteins demonstrated increased abundance in the post-nebulized samples (Table 2). Differences in peptide abundances were detected between the pre- and post-nebulized samples with 54/2,890 quantifiable peptides significantly increased in abundance in the pre-nebulized sample (relative to post-nebulized) and 142/2,890 quantifiable peptides significantly decreased in the post-nebulized samples (relative to pre-nebulized; Supplementary Figure 1).

**Table 2:** Proteins identified through digestion of platelet lysate that are increased (≥1.5) or decreased (≤0.5) in abundance in the pre-nebulized sample as compared to post nebulized samples (*n* = 3).

**Protein**	**Ratio**
**Pre/post** ≥**1.5**	
Adiponectin A	1.5
Apolipoprotein E	1.5
RAP1β, member of RAS onco family	1.8
Nucleosome assemble protein 1 like 1	2
Phosphoribosyl pyrophosphate synthetase associated protein 2	2.3
Alpha-1-antitrypsin	2.4
Ig-like domain-containing protein	2.5
Phospholipase A2 group VII	5.3
**Pre/post** ≤ **0.5**	
Tubulin beta chain	0.45
Transferrin receptor protein 1	0.37
Tyrosine 3-monooxygenase/tryptophan 5-monooxygenase activation protein epsilon	0.36
Glutamate receptor	0.33
Phosphogluconate dehydrogenase	0.32
Cysteine-rich secretory protein 3	0.31
Lymphocyte cytosolic protein 1	0.28
BUB1 mitotic checkpoint serine/threonine kinase	0.19
F-actin-capping protein subunit beta	0.14
Phospholipid transfer protein	0.09
Inter-alpha-trypsin inhibitor (Fragment)	0.07
Adenine phosphoribosyltransferase	0.05

### 3.4 Bacterial growth curve assay

**Figure 4:**
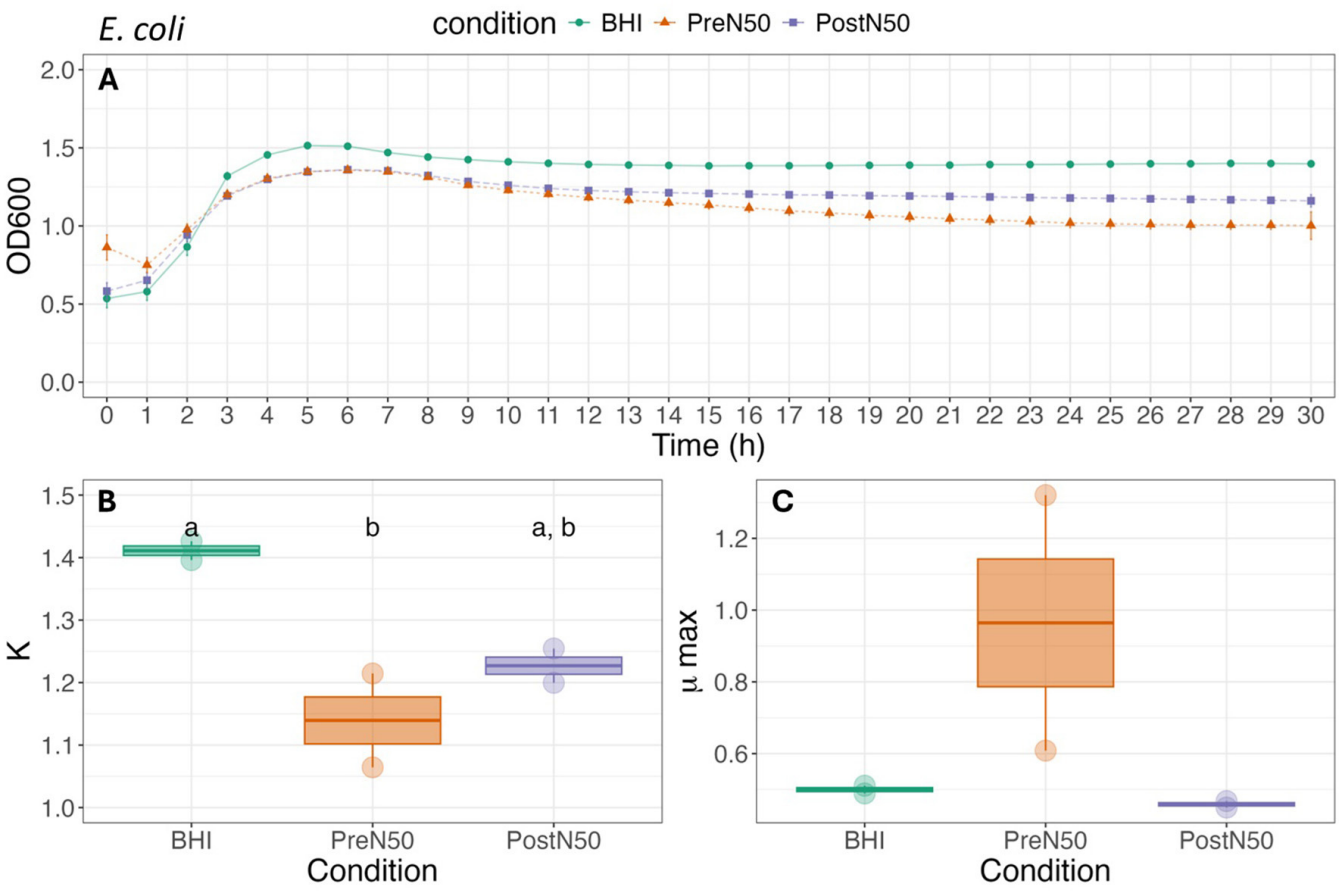
Growth curve **(A)**, carrying capacity (K, h^−1^) **(B)** and maximum growth rate during exponential growth (μmax, OD_600_) **(C)** of *E. coli* under different conditions. For **(B, C)**, different lower-case letters represent statistically significant effect (with BHI as the reference group) or difference among groups at *P* ≤ 0.05 using linear modeling and *post-hoc* tests (Tukey HSD test). Single pre- and post-nebulized aliquots used for analysis.

**Figure 5:**
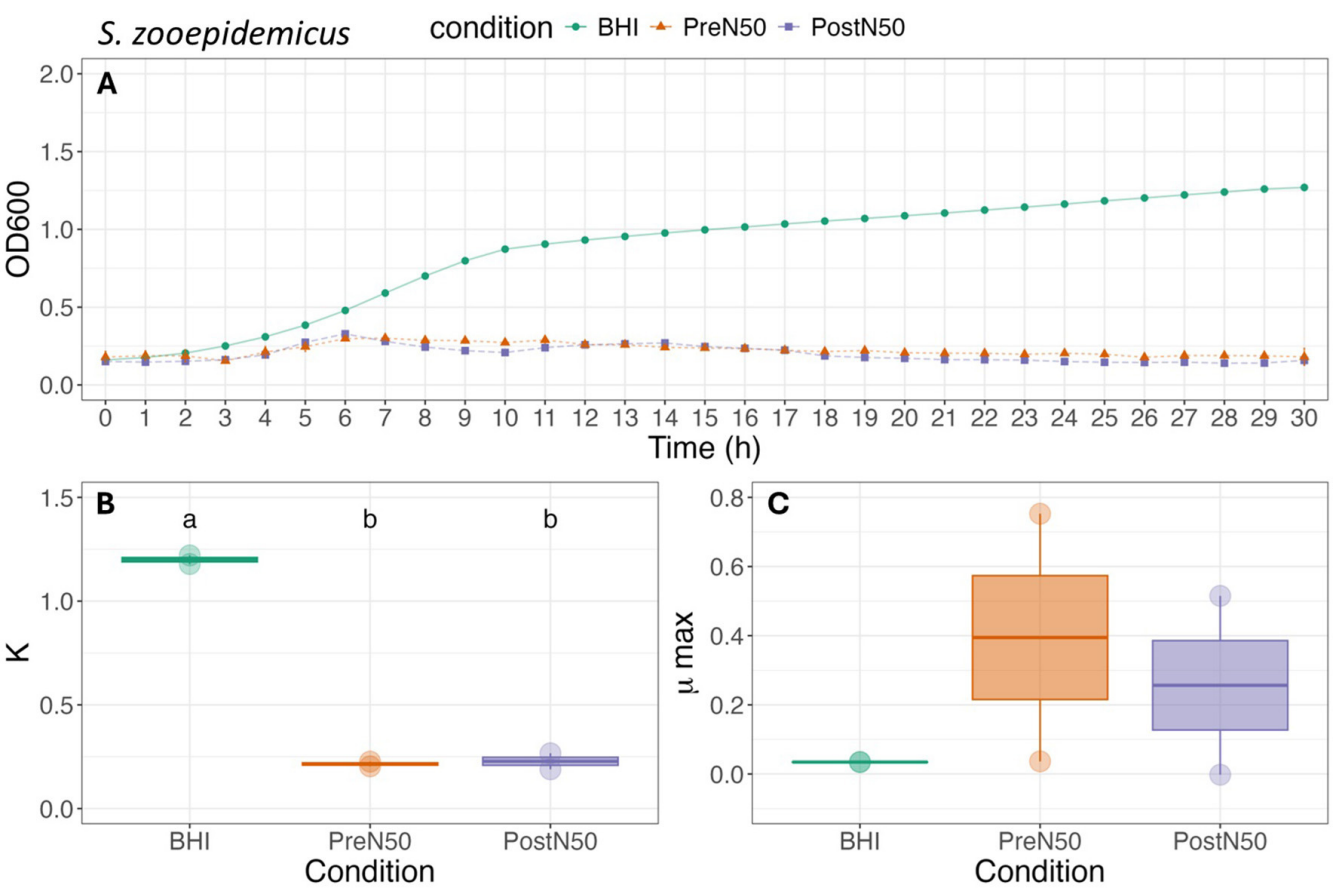
Growth curve **(A)**, carrying capacity (K, h^−1^) **(B)** and maximum growth rate during exponential growth (μmax, OD_600_) **(C)** of *S. zooepidemicus* under different conditions. For **(B, C)**, different lower-case letters represent statistically significant effect (with BHI as the reference group) or difference among groups at *P* ≤ 0.05 using linear modeling and *post-hoc* tests (Tukey HSD test). Single pre- and post-nebulized aliquots used for analysis.

**Figure 6:**
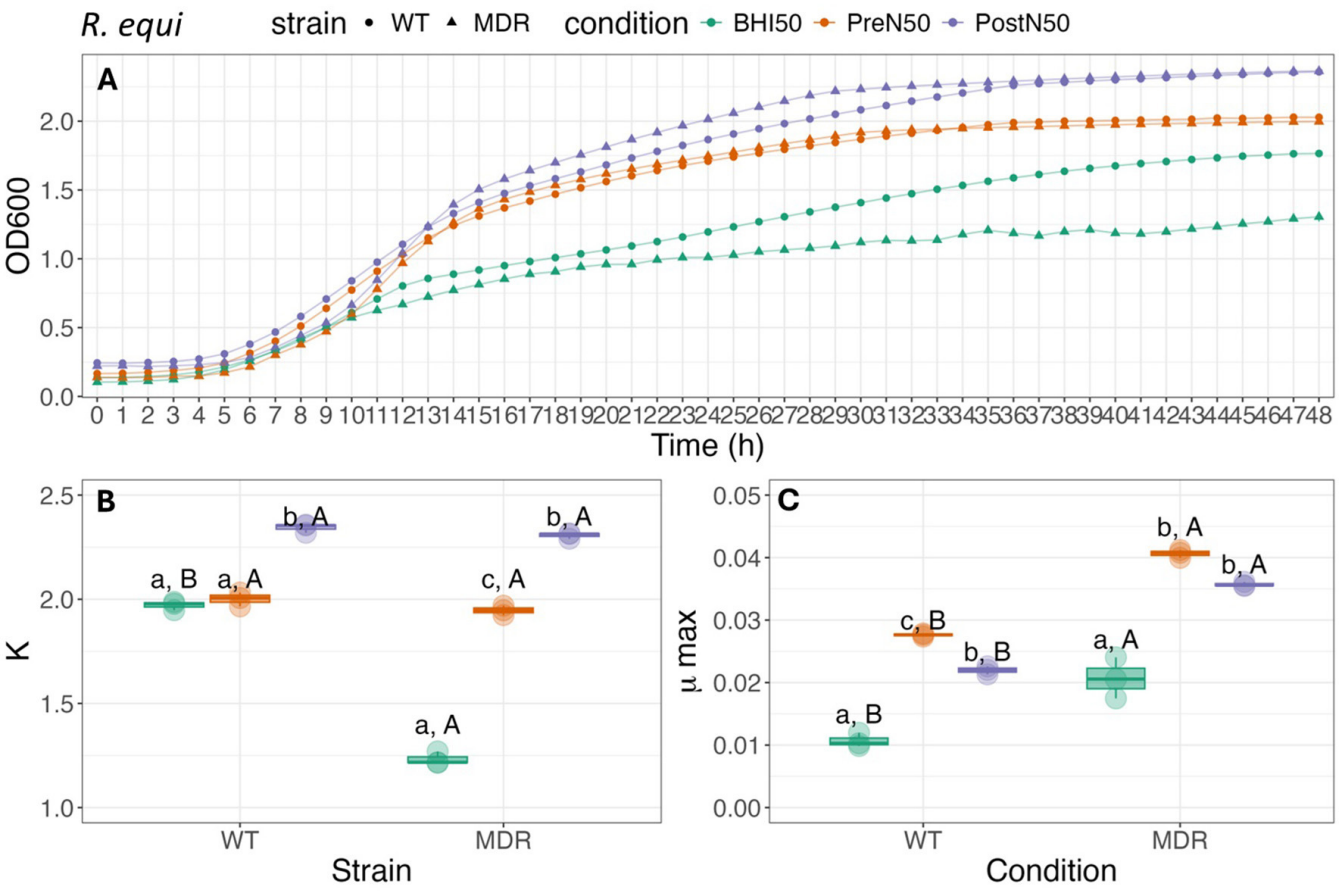
Growth curve **(A)**, carrying capacity (K, h^−1^) **(B)** and maximum growth rate **(C)** during exponential growth (μmax, OD_600_) of WT*-R. equi and* MDR*-R. equi* under different conditions. For **(B, C)**, different lower-case letters represent statistically significant difference among conditions within the same strain; different capital letters represent statistically significant difference between strains for the same condition at *P* ≤ 0.05 using Tukey HSD test. Single pre- and post-nebulized aliquots used for analysis.

Growth curves and associated carrying capacity (K) and maximum growth rate during exponential growth (μmax) for *E. coli, S. zooepidemicus*, and *R. equi* (WT, MDR) are depicted in Figures 4–6.

#### 3.4.1 Nebulization does not change the effects of PL on bacterial growth rate parameters of *E. coli* and *S. zooepidemicus*

As illustrated in Figure 4, differences in K and μmax of *E. coli* were not observed between the pre- (PreN50) and post-nebulization (PostN50) treatments. Addition of PreN50 decreased carrying capacity (K) when compared with the BHI control (*p* = 0.026). However, *post-hoc* tests revealed that carrying capacity did not differ among groups. No effect of condition (treatment or control) was observed on μmax of *E. coli*.

As illustrated in Figure 5, differences between the PL treatments were not observed for K or μmax of *S. zooepidemicus*. The addition of PL decreased carrying capacity (K) of *S. zooepidemicus* when compared to the BHI control (*p* = 0.009) for both the PostN50 (*p* = 0.046) and PreN50 (*p* = 0.043) treatments. No effect of PL treatment was observed on the μmax of *S. zooepidemicus*.

#### 3.4.2 Pre- and post-nebulized PL effect bacterial growth rate parameters for both strains of *R. equi* (WT and MDR)

As illustrated in Figure 6, K of WT-*R. equi* was higher for PostN50 when compared to both the PreN50 treatment and BHI control (*p* ≤ 0.001). There was no effect of treatment when PreN50 and BHI were compared. For MDR-*R. equi*, K was higher for both PL treatments when compared to the BHI control (*p* < 0.001). K was higher in PostN50 compared with PreN50 (*p* < 0.001).

Maximum growth (μmax) of WT-*R. equi* was higher when grown in PreN50 compared to PostN50 (*p* = 0.036) and for both PL treatments when compared with BHI (*p* < 0.001). Maximum growth (μmax) of MDR- *R. equi* did not differ between PL treatments (*p* = 0.38). The μmax of both PreN50 and PostN50 was higher compared with the BHI control (*p* < 0.001).

When the WT and MDR *R. equi* strains were compared, K was higher for WT-*R. equi* (*p* < 0.001) when grown in BHI. Differences between strains in K were not observed for WT-*R. equi* and MDR-*R. equi* grown in PostN50 (*p* = 0.75) or PreN50 (*p* = 0.25). When comparing between strains, MDR-*R. equi* had higher μmax compared with WT-*R. equi* in all media types (*p* = 0.035, *p* < 0.001, *p* < 0.001, for BHI, PreN50, and PostN50, respectively).

## 4 Discussion

In this present pilot study we evaluated *in vitro* the feasibility of administering equine platelet lysate using an equine specific vibrating mesh nebulizer. To the best of our knowledge, similar studies have not been performed. Additionally, while not a primary objective, this study is also unique in describing the proteomic profile of pooled equine platelet lysate manufactured via plateletpheresis. In agreement with our study hypotheses, clinically relevant flow rates and particle size distribution were achieved. A limited number of growth factor and cytokines demonstrated a change in concentration >10% and some differences in protein expression were identified in response to nebulization in the proteomic profile. Assessment of antimicrobial activity against *E. coli, S. zooepidemicus*, and *R. equi* demonstrated similar effects of equine PL on several bacterial growth inhibition parameters before and after nebulization; however the effect of PL and nebulization on these parameters differed by bacterial species.

Inhalation therapy, including via nebulization, is used with increasing frequency in horses for treatment of equine asthma and pneumonia (39, 40). Achievable flow rates and particle size distribution are important factors to consider when determining the suitability of a medication for nebulization. Considering patient tolerance and compliance, nebulization time for horses should be as short as possible, ideally not exceeding 20–25 min (40). At our measured flow rate of 0.8 ml/min using the Flexineb^®^ nebulizer system equipped with the Standard Flow medication cup, this would allow for delivery of 16–20 mL of lysate. While predicting a therapeutic dose for any inhaled medication can be challenging and remains to be determined for nebulization of PL, these volumes fall within the range of 10–20 ml reported for direct intrabronchial instillation of PRP in adult horses with EIPH (29) or equine asthma (28). Aerosolized particle size distribution is a key factor determining appropriate deposition of an aerosolized medication within the airways and thus its' relevance for use in treating infectious or inflammatory conditions of the lung (39, 40). In this study, 52% of aerosolized PL particles were measured to be ≤ 5 μm and 85% of particles to be ≤ 10 μm in size. Aerosolized particles 1–5 μm in size are deposited in the smaller bronchioles and alveoli and are generally considered therapeutic aerosols (31, 41). Particles 6–10 μm may still hold relevance for treatment of conditions such as pneumonia because they are deposited in the larger bronchioles and bronchi (41). Thus, although factors beyond particle size can influence aerosol penetration in a live animal, our results suggest that nebulized PL could reach targeted areas for treatment of pneumonia.

Aerosol generation for mesh nebulizers such as Flexineb^®^, occurs when high frequency vibrations drive liquid through small pores of a metallic mesh membrane located within a medication cup (31, 39, 42). The flow rate and distribution of particle sizes are primarily determined by pore size and the penetration of solution through the pores (31, 39). Alternative nebulizer systems used in horses include jet (compressor) and ultrasonic nebulizers. The flow rate and particle size distribution reported in the present study should not be assumed to be the same for other types of nebulizers due to differences in function or performance among these systems (31, 39, 42). Regardless of nebulizer type, consideration must be given to the viscosity of the solution undergoing nebulization. Subjectively, equine PL is viscous. This is not unexpected given that the viscosity of equine plasma is greater than other species and is approximately 1.5 times that of saline (19, 43). For mesh nebulizers, viscous solutions can clog pores in the mesh of the medication cup, reducing flow rate and potentially altering particle size distribution (31, 33, 39). The flow rates reported in the present study were achieved using a Standard Medication cup which is most often utilized in clinical practice. Delivery of larger volumes of PL in a clinical setting could potentially reduce effectiveness of nebulization due to viscosity. A medication cup with increased pore size (Fast Delivery, Flexineb^®^) is available to accommodate more viscous solutions. Unfortunately, while flow rate is improved, aerosolized particle size is increased (33, 39), potentially impacting deposition within the lung. Lyophilization of equine platelet extracts appears to preserve biological activity while allowing for reconstitution in water (44) and could be evaluated in future studies for suitability for nebulization.

For this study, we chose to evaluate concentration changes in response to nebulization for select growth factors (PDGF, TGF-β1), pro- (IL-1β, IL-6, TNF-α, IFN-γ) and anti-inflammatory (IL-4, IL-10) cytokines, and the antimicrobial peptide (Beta Defensin-1), that could hold potential relevance for the use of PL in treating bacterial pneumonia. Variability across studies in reported composition and activity of these bioactive molecules within equine PL is most likely due to differences in experimental design as well as inherent differences among donor populations (10, 13, 45, 46) and in methods of processing and preparation of PL (10, 12, 47–49). Additionally, while the impact of storage time at −80°C on concentrations of growth factors or other proteins has not been described for equine PL, variable changes over time in concentration of some growth factors have been reported for equine and human PRP stored at −80°C (50, 51). Overall, growth factor and cytokine concentrations in this study were slightly lower or comparable to those previously reported for equine PL products (13, 48, 49, 52, 53). Concentrations of all measured effector molecules except IL-4 were decreased after nebulization. It is unknown whether these changes, if repeatable, would carry clinical significance. The pro-inflammatory cytokine, IL-1β, demonstrated a 20% reduction post-nebulization. Increases in IL-1β gene expression have been detected in BAL fluid in response to inflammatory conditions, including in horses with severe equine asthma (54, 55) and humans with community-acquired pneumonia (56). Interestingly, nebulization of human platelet lysate via jet nebulizer (22), resulted in mild increases in PDGF and TGF-β1 concentrations and this was attributed to water evaporation during the nebulization process. Nebulization of a larger number of replicates or from different sources of PL would have provided a more robust assessment regarding whether the concentration changes observed in this study represent true degradation of PL in response to nebulization. Additionally, cell culture studies could have allowed for evaluation of potential changes in cytokine or growth-factor mediated bioactivity after nebulization.

Proteomic analysis was performed on PL before and after nebulization to provide a more comprehensive assessment regarding the impact of nebulization on protein composition. Thermal and mechanical stresses that occur during the process of nebulization can potentially alter the stability of macromolecules and lead to protein degradation particularly within high protein solutions, such as PL (31). While these stresses are of greatest concern with ultrasonic nebulization, they may still be of relevance to mesh nebulization (31). Complementary methods of protein digestion and low molecular weight filtration were chosen for proteomic analysis in this study with the goal to improve detection of smaller proteins and peptides that could be missed with digestion alone. Proteomic profiles are lacking for equine PL but have been utilized to highlight differences in human PL according to methods of preparation (57–61). Variability in the methodological approach to proteomic analysis makes it difficult to draw comparisons not only with the proteomic profile described in this study, but also among the referenced human PL studies. Nevertheless, some overlap in the most common biological processes identified through GO analysis exists between this study and what was reported by Le et al. (57) for human PL.

Observed differences in abundance and expression of proteins and peptides between pre- and post-nebulized PL samples were quite small. The peptide, beta defensin 3, was the only peptide (or protein) uniquely expressed in pre-nebulized platelet lysate. Interestingly, this peptide could be of relevance for treatment of bacterial pneumonia. Beta defensin 3 is one of a group of low molecular weight (< 10 kDa) cationic peptides with constitutive or induced expression and that demonstrate antimicrobial and immunomodulatory activity in a variety species (60–62). While it is possible this peptide was lost or degraded during the process of nebulization, given the small size (5 kDa), it is also possible that lack of detection in the post nebulization samples is secondary to sample processing prior to digestion or LC/MS analysis. This may be a more reasonable explanation as thymosin β-4, another antimicrobial peptide of similar molecular weight, was identified in pre- and post-nebulized samples, and the related peptide, beta defensin 1, was identified using ELISA in similar concentrations in both pre- and post-nebulized samples.

The impact of nebulization on the antimicrobial bioactivity of equine PL was evaluated in this study through use of a bacterial growth curve assay. *E. coli, S. zooepidemicus* and *R. equi* (WT and MDR) strains were chosen because of their relevance to bacterial pneumonia in adult and juvenile horses (1, 2, 4). Although 100% platelet lysate would most likely be used in a clinical setting (12, 63–65), for this study PL was diluted to 50% in BHI to allow distinction of growth inhibition due to direct effects of PL vs. due to a lack of nutrients, as has been demonstrated when >60% PL is used in growth inhibition assays (12, 16). Overall, any differences in antimicrobial activity of PL observed in this study and associated with nebulization were small and largely limited to *R. equi*. For both the WT and MDR *R. equi* strains, growth rate parameters increased with addition of PL irrespective of nebulization. This effect was more prominent for maximum growth rate (μmax) with pre-nebulized PL and for carrying capacity (K) with post-nebulized PL.

The antimicrobial activity of PL and other platelet derived products is a complex process that has been demonstrated for a variety of Gram negative and positive bacteria (10, 12, 20, 21, 46, 66–70). The effects of PL on *in vitro* bacterial growth for *S. zooepidemicus* observed in this study is consistent with a previous study that demonstrated equine PL antimicrobial activity against *S. zooepidemicus* biofilms (10). In our study, growth inhibition of *E. coli* when exposed to PL was minimal. The antimicrobial activity of equine PL against *E. coli* is inconsistently reported across studies (10, 12, 16). Using PL prepared as described in this study, Gordon et al. (12) demonstrated reduced growth rate, delayed growth, and lower bacterial yield for *E. coli* when exposed to similar concentrations of PL. Explanations for the discrepancy between our results and those reported by Gordon et al. (12) could include inherent differences in susceptibility between strains of *E. coli* or in expression of specific bioactive molecules between PL batches, or potentially in differences in experimental protocols.

To the best of our knowledge, this study is the first to investigate antimicrobial activity of equine PL against *R. equi*. The overall effect of PL increased *R. equi* growth relative to BHI in this study were unexpected but should be interpreted with caution as they do not necessarily indicate that PL supports growth of *R. equi*. Detailed *in vitro* characterization of the antimicrobial effects of PL on *R. equi* was beyond the scope of this pilot study but should be performed to determine whether PL is contraindicated with *R. equi* pneumonia. As demonstrated for other bacteria, the specific antimicrobial effects of PL can be unique to bacterial strain both within and across studies and can potentially vary according to methods used for preparation of PL and evaluation antimicrobial activity or potentially other experimental factors (46). For example, the concentrations and activity of cytokines, growth factors and peptides expressed in PL and important for antimicrobial and related immunomodulatory activity can be influenced by donor pool (13), number of freeze thaw cycles (47, 57–59), and other methods of preparation such as fractionation to increase concentration of low molecular weight cationic peptides (20). Improved antimicrobial activity has also been demonstrated through co-administration of PL and an antibiotic (20). Furthermore, *in vitro* evaluation does not account for potential indirect effects of growth factors, cytokines, or antimicrobial peptides within PL on cellular and immunomodulatory actions that could play critical roles in influencing antimicrobial activity against *R. equi* through promoting phagocytosis, T-cell stimulation, or activation and recruitment of other immune cells (62, 71, 72).

There are limitations to this pilot study. The nature of this study is *in vitro*, and our findings may not reflect deposition, anti-inflammatory and/or antimicrobial activity of PL in live horses with respiratory disease where changes in breathing pattern, increased respiratory secretions, and damage to the airways can all impact delivery and activity of aerosolized medications (40). Aerosolized delivery of medications can potentially cause irritation to the airways and thus nebulization of PL to healthy horses followed by cytological evaluation of bronchoalveolar lavage fluid would provide assessment of tolerance to nebulization and would also add valuable information regarding any pro-inflammatory or other undesirable affects within the airways. Assessment of antimicrobial activity was limited to growth curve analysis with OD_600_ readings used to approximate the number of bacteria present in the sample. Over estimation of the number of bacteria can occur with OD readings since they do not distinguish between live and killed bacteria (12). Moreover, experimental error is possible with this kind of approach, demonstrated by the overestimation of *E. coli* concentration in the beginning of the experiment. Because the concentration was adjusted in the first hour after that and followed the expected concentration for each experimental time, repeating the measurements was not justified. Additional approaches that may have provided a more thorough characterization of the antimicrobial effects of PL could have included evaluation using killing assays, broth microdilution or disk diffusion methods both with or without co-culture with relevant antimicrobials. Finally, although PL used in the study was pooled, only three horses were used for preparation and evaluation of the effects of nebulization relied on technical replicates from a single batch. Increasing the number of biologic replicates by including additional batches of PL as well as increasing the number of horses included in the pooled lysate may have provided a more robust characterization of cytokine, growth factor, and protein composition and antimicrobial activity before and after nebulization.

## 5 Conclusion

In conclusion, the results of this pilot study demonstrate that nebulization of equine PL is technically feasible using a commercially available equine mesh nebulizer based on determination of particle size and flow rate. In addition, *in vitro* evaluation of nebulized PL resulted in some changes in protein composition and antimicrobial properties with the antimicrobial effects of PL being organism specific. Further investigation is warranted to better define *in vitro* the potential antimicrobial and anti-inflammatory properties of nebulized equine PL with the goal to ultimately determine its therapeutic potential in horses with bacterial pneumonia or other inflammatory airway conditions such as equine asthma.

## Data Availability

The datasets presented in this study can be found in online repositories. The names of the repository/repositories and accession number(s) can be found in the article/Supplementary material.
