# Supplementary figures and images for: Pilot study characterizing a single pooled preparation of equine platelet lysate for nebulization in the horse
Source: Front Vet Sci. 2024 Dec 12;11:1488942. doi: 10.3389/fvets.2024.1488942 (PMC11670369; doi:10.3389/fvets.2024.1488942)

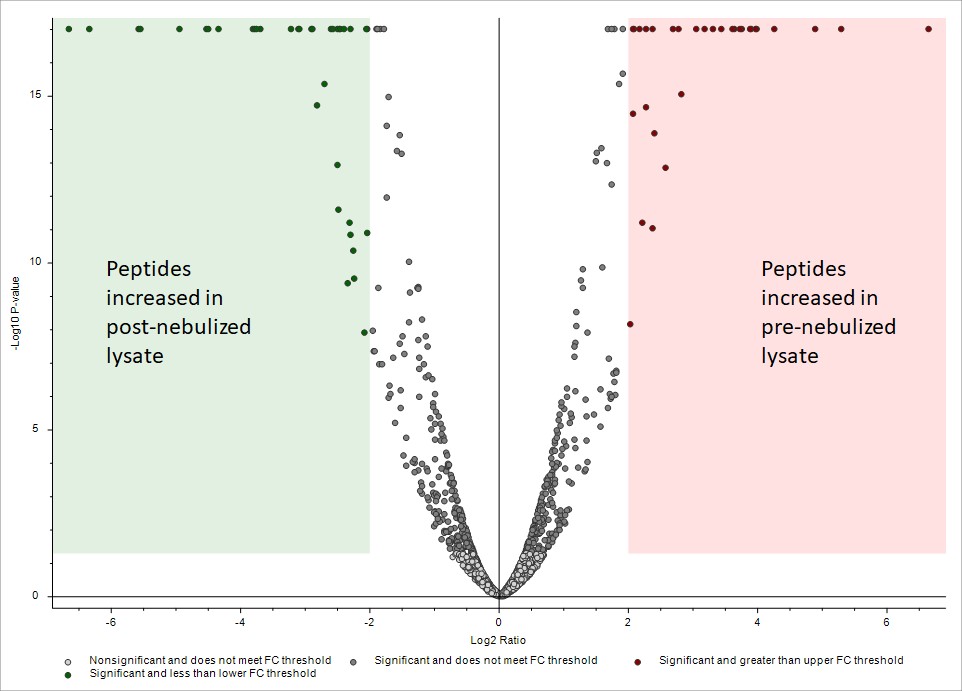

Supplement: Supplementary file 1 [file Image_1.JPEG]

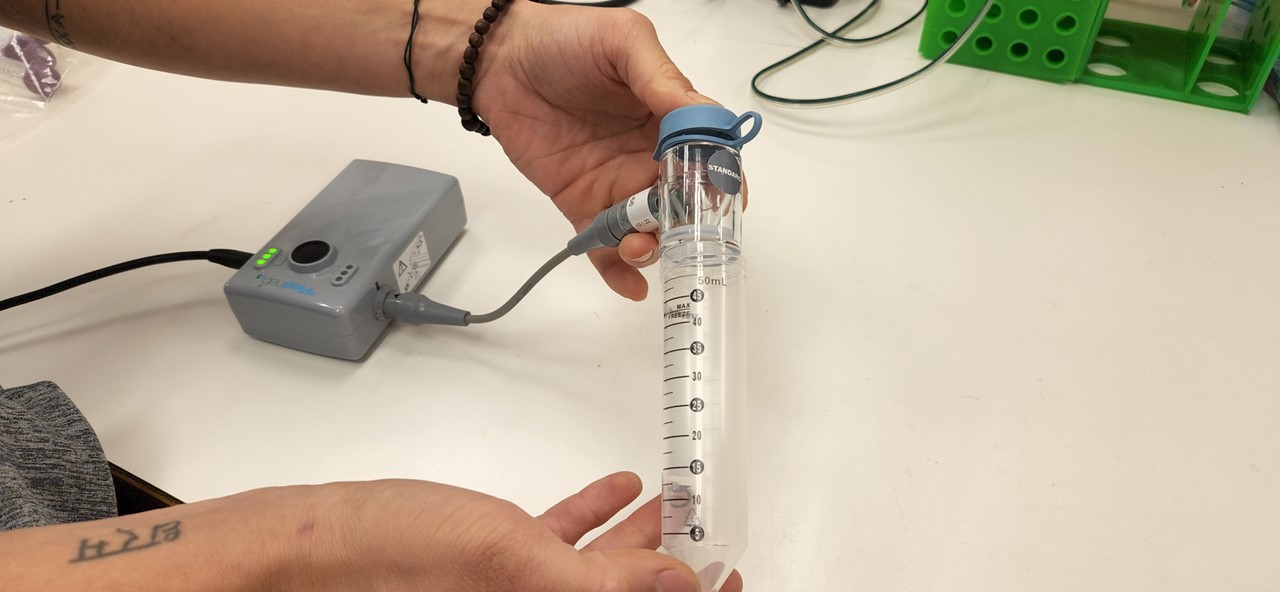

Supplement: Supplementary file 2 [file Image_2.JPEG]
