# Supplementary material for: Pilot study characterizing a single pooled preparation of equine platelet lysate for nebulization in the horse
Source: Front Vet Sci. 2024 Dec 12;11:1488942. doi: 10.3389/fvets.2024.1488942 (PMC11670369; doi:10.3389/fvets.2024.1488942)
Supplement: Supplementary file 4 [file Table_2.DOCX]

Supplementary Material

# Supplementary Data

# Supplementary Figures and Tables

## Supplementary Figures

**Supplementary Figure 1.** Volcano plot of **peptide abundances** pre vs post nebulization. A total of 3235 peptides were identified, of which 54 peptides were significantly increased in abundance (red) and 142 peptides were significantly decreased in abundance (green) in the pre-nebulized sample relative to the post nebulized samples. This plot uses a log2 fold change cutoff of 2 and a p value cutoff of 0.05.

**2.2 Supplementary Tables**

**Supplementary Table 1:** Proteins identified in pre- and post-nebulized platelet lysate through both digestion and MWCO filtration (n = 65; green shading), through only protein digestion (n = 219; blue shading) or through only MWCO filtration (n = 172; orange shading).
